# Supplementary material for: Prevalence of Antibodies to Japanese Encephalitis Virus and Severe Fever with Thrombocytopenia Syndrome Virus in Wild Boars Captured Across Different Locations in Toyama Prefecture, Japan
Source: Viruses. 2025 Dec 5;17(12):1585. doi: 10.3390/v17121585 (PMC12737401; doi:10.3390/v17121585)
Supplement: Supplementary file 1 [file viruses-17-01585-s001.zip › viruses-3969735-supplementary.pdf]

Supplementary Material

Yazawa *et al*., Supplementary Fig.1A

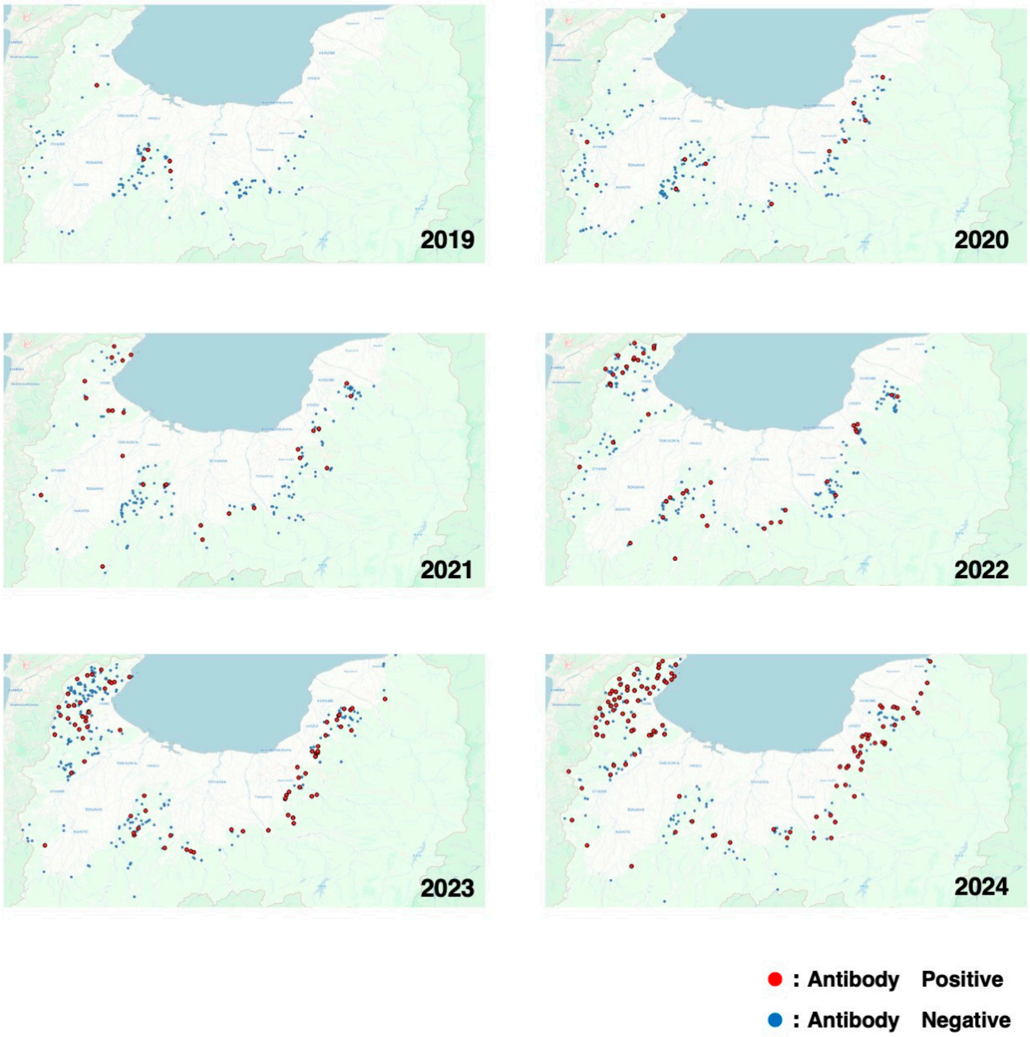

Yazawa *et al.*, Supplementary Fig.1B

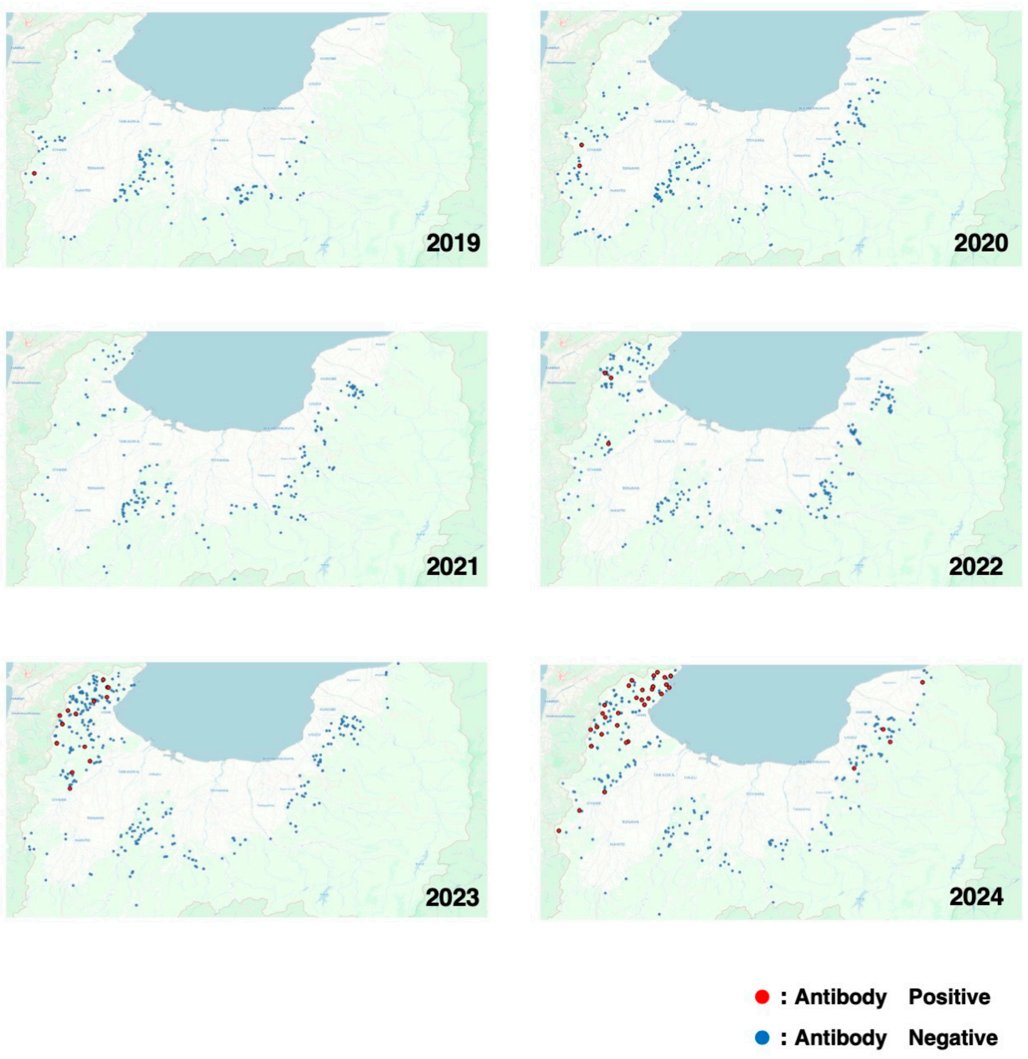

Supplementary Figure S1. Annual geographical distribution of antibody-positive wild boars. (A) JEV survey and (B) SFTSV survey. Red dots indicate capture locations of antibody-positive wild boars, while blue plots indicate capture locations of antibody-negative wild boars. Each figure panel is labeled with the corresponding year in the bottom right corner.
